# Visual modelling can optimise sticky trap design for simultaneous monitoring of multiple species of insect pests

**DOI:** 10.1038/s41598-025-01954-8

**Published:** 2025-05-19

**Authors:** Natalie S. Roberts, Jean Claude Ndayiragije, Tuğçe Özek, Tariq M. Butt, İsmail Karaca, Farooq Shah, William L. Allen

**Affiliations:** 1 Department of Biosciences, Faculty of Science and Engineering, Swansea University, Swansea, SA2 8PP UK; 2 Department of Biology, Lund University, 223 62 Lund, Sweden; 3 Department of Plant Protection, Faculty of Agriculture, Isparta University of Applied Sciences, 32260 Isparta, Turkey; 4 100/2000 CoHE PhD Scholarship, Isparta, Turkey; 5 Enterprise Centre, Razbio Limited, Bryn Road, Bridgend, CF32 9BS UK

**Keywords:** Vision, Integrated pest management, *Frankliniella occidentalis*, *Trialeurodes vaporariorum*, Thrips, Whitefly, Colour vision, Animal behaviour, Computer modelling

## Abstract

Coloured sticky traps are commonly used to monitor insect pests. Colour affects trap performance, with preferred colours often differing between species, making selection of trap colour for effective management of multiple pests challenging. Greenhouse whitefly (GWF) *Trialeurodes vaporariorum* and Western flower thrips (WFT) *Frankliniella occidentalis*, are major horticultural pests that often co-occur. Yellow colours are attractive to GWF, while blue is often used to target WFT, although WFT are also attracted to yellow colours. The visual mechanisms that make yellow colours attractive to either species are poorly understood. Previous experiments in WFT find that visual modelling of an opponent mechanism between short wavelength sensitive (SWS) and long wavelength sensitive (LWS) photoreceptors optimises the performance of blue sticky traps. In the current study, we assess whether an opponent response that highly stimulates LWS relative to SWS photoreceptors predicts the attractiveness of yellow sticky cards to both WFT and GWF. Our results showed that yellow sticky cards that maximize a predicted SWS:LWS opponent mechanism improves capture for both species. Further, optimising the SWS:LWS ratio allowed for simultaneous monitoring of both pest species using single colour cards. We also showed that sticky trap colour and luminance are comparable across different lab and field contexts, highlighting the broad applicability of visual modelling in pest management strategies.

## Introduction

Coloured sticky traps are a common method for monitoring and control of insect pests. Hundreds of studies have assessed the effectiveness of different colours for different types of pests, typically by measuring capture rate for different colours of sticky trap^1^. The most attractive colour or colours often differ based on species^2^. Even within species, differences in colour attractiveness have been reported based on sex, population, or surrounding environment^3–5^. This variation between and within species in colour attraction is a challenge for pest monitoring and control when multiple species are targeted, and environmental conditions vary. Using multiple trap types with different colours increases the costs of deploying, collecting, and analysing trap capture for effective pest management. Thus, there is a need for traps that can optimally target multiple pest types.

The greenhouse whitefly (GWF), *Trialeurodes vaporariorum*, and the Western flower thrips (WFT), *Frankliniella occidentalis,* are two common insect pests. GWF and WFT are horticultural pests that are present worldwide, including in greenhouses and polytunnels. They cause direct damage to plants via feeding and oviposition damage, and indirect damage via transmission of plant pathogens^6–9^. As they are often active in similar crops at similar times^10,11^ growers monitor for both pests simultaneously to assess crop risk and respond appropriately. Studies of colour preferences for GWF show that yellow-coloured traps are generally most attractive^12,13^. In WFT, blue sticky traps are often found to be most attractive, and yellow colour generally the next most attractive, however, in some studies yellow seems to be preferred^14^. These differences in colour preferences between GWF and WFT means that multiple sticky traps are used to monitor separately for each pest species. However, the secondary attractiveness of yellow to WFT provides the opportunity for a single colour sticky trap for monitoring of multiple species. Yellow sticky traps are already used for broadcast monitoring of arthropods^2,15^, however it is unclear what factors impact the performance of yellow sticky traps for either GWF, WFT, or other insects, including beneficial insects. The gap in understanding of why certain colours are preferred and what qualities of trap colour impact performance limits our ability to effectively employ coloured traps for pest monitoring. An obvious shortcoming of previous studies on colour preference in pest control are that they do not consider colour from the perspective of the insect model, with differences between human and insect visual systems likely reducing our ability to exploit visually guided insect behaviours^16–18^.

Previous studies have found that accounting for the visual system of target species via visual modelling can improve design of colour traps^19–23^. Modelling techniques can use information on aspects of visual performance to predict the visual response of target pests to different colour traps^16^. Two key components to predicting visual response to stimuli are the spectral sensitivity of photoreceptor classes present in the visual system and the subsequent processing of photoreceptor outputs, such as colour opponent visual processing. Colour opponent mechanisms involve comparing the relative excitation ratios between combinations of two or more photoreceptor classes to assess the chromatic properties of the stimulus independent of achromatic properties. Studies in GWF and WFT suggest opponent processing occurs between photoreceptor classes present in the visual system^24,25^. GWF and WFT possess three photoreceptor types, an ultraviolet sensitive (UVS), short-wavelength sensitive (SWS), and long-wavelength sensitive (LWS) receptor, with maximum sensitivity to the UV, blue, and green part of the visual spectrum, respectively. Opponent processing between the blue SWS and green LWS receptors affects wavelength-specific settling responses in both species, albeit in opposite directions, with blue light (λ_max_ = 467) being the most attractive to WFT and the most inhibitory to GWF^24,25^. In WFT, modelling of the hypothesized blue-green (BG) opponent mechanism, substantially improved trap performance relative to current commercial blue traps. Namely, traps that predicted high stimulation of WFT blue-sensitive SWS receptors relative to the green-sensitive LWS receptor caught significantly more WFT than traps with a lower opponent signal^19^. Because photoreceptor stimulation can be reliably calculated using modelling approaches common in sensory ecology research^16,17^, this study demonstrates how integration of visual modelling in pest management strategies can exploit known properties of insect vision to impact pest behaviour.

Visual modelling can also be used to investigate other aspects of trap appearance that may affect performance such as perceived lightness, which includes achromatic information relating to the intensity or total reflectance of a stimulus. For example, visual models based on behavioural data from the tsetse fly *Glossina fuscipes fuscipes* suggest that both chromatic and achromatic channels impact performance of coloured traps^26^. Behavioural studies using LED lights show that increasing the intensity of blue LEDs for WFT and yellow LEDs in GWF increases stimuli attractiveness^25,27^. Further, there appears to be an interactive effect of colour and luminance in GWF, such that increasing the brightness of typically less attractive colours relative to a preferred colour resulted in a switch in colour preference^25^. These potentially interacting effects of chromatic and achromatic information have not been thoroughly investigated in the context of non-luminous coloured sticky traps, which may lead to confounding results in tests of colour performance if various visual properties of colour are not considered^28^.

In the current study, we aim to test whether a BG opponent response and/or luminance impacts attractiveness of yellow colours and whether accounting for these visual properties can facilitate efficient design of yellow traps that are more effective for multiple pest capture, or capture targeted to specific species. We use visual modelling to predict quantal catch for the three WFT and GWF photoreceptor classes to colours spanning yellow-green to orange-red hues. We estimate the opponent response for the hypothesised BG opponent mehcanism^24,25^ and assess trap luminance as perceived by WFT and GWF, to determine how BG ratio (chromatic cues) and luminance (achromatic cues) independently or interactively affect attraction to traps in field trials (experiment 1). Further, we directly test whether visual modelling can maximise simultaneous capture of both target species by quantifying the effectiveness of traps varying in chromatic and achromatic properties on multi-pest capture (experiment 2).

## Methods

### Colour swatch creation and photography

Methods for colour swatch creation and photography generally followed methods described in Dearden et al.^19^. Briefly, we generated swatches of potential trap colours that varied in colour in Adobe Photoshop (Adobe Inc., USA) by changing RGB values for target colours. Colour swatches were printed on Koala Photo Satin 190 GSM paper using a Canon Pro 4100S printer. Sticky insect glue (Horsefly Glue, Sticky-trap Ltd., UK) was applied uniformly over the paper surface prior to photography.

We photographed colour swatches to measure quantal catch for GWF and WFT used for calculation of BG ratio and trap luminance. Colour swatches were photographed in July 2023 (experiment 1) and August 2023 (experiment 2) at Swansea University (Swansea, UK) in direct sunlight using a Samsung NX 1000 camera following previously described methods^19^. The camera was modified to remove the built-in UV filter, allowing us to measure UV reflectance from photographs. Because the camera sensors do not inherently differentiate between UV and visible light, we used a BAADER U-Filter (Baader Planetarium GmbH, Germany), which transmits wavelengths between 320 and 380 nm, and a BAADER UV/IR-Cut/L, which transmits lights between 400 and 680 nm, to take separate UV and visible spectrum photographs. Each colour swatch was photographed twice, once with the UV and again with the visible spectrum filter and the resulting RAW UV and visible photographs were then manually aligned using the micaToolbox plugin for ImageJ to generate a multispectral (.mspec) image^29,30^.A 2% and 75% reflectance standard (Labsphere Inc., UK) were also included in photographs.

### Visual modelling

To generate spectral sensitivity curves, we used a custom MATLAB script to predict photoreceptor sensitivity following a Govardovskii template^31^ using previously recorded λ_max_ values for each species. Behavioural experiments in GWF suggest the presence of three visual pigments, with λ_max_ = 360 nm (UVS), 480 nm (SWS), and 515 nm (LWS)^25^. Electroretinogram (ERG) and behavioural experiments in WFT also suggest three visual pigments, with λ_max_ = 363 nm (UVS), 476 nm (SWS), and 535 nm (LWS)^24^. After generating sensitivity curves for each species, quantal catch of the UVS, SWS, and LWS photoreceptors was measured for each swatch colour photographed using the micaToolbox and ImageJ.

We calculated the BG ratio and luminance of each colour swatch using quantal catch measurements for GWF (λ_max_ UV = 360 nm, SWS = 480 nm, LWS = 515 nm) and WFT (λ_max_ UV = 363 nm, SWS = 476 nm, LWS = 535 nm). BG ratio was calculated using the following formula: BG ratio = (B-G)/(B + G), where B = blue (SWS) photoreceptor excitation and G = green (LWS) photoreceptor excitation^19^. Because we focus on colours in the yellow part of the visual spectrum, BG ratios that are more negative (i.e. further from zero) represent a stronger opponent response. Luminance was calculated as quantal catch from the LWS photoreceptor normalised against the reflectance standards ^29,32^.

We next calculated the chromatic and achromatic perceptual distances between colour swatches using a receptor noise limited model^33^, which gives the number of just noticeable differences (JNDs) between each colour swatch. JNDs represent the smallest visual difference that can be perceived, with JNDs < 1 being considered below threshold, although JNDs < 3 are often considered to be poorly discriminable when considering visual performance in natural lighting conditions^34,35^. Quantal catch estimates for each colour swatch from micaToolbox were imported into the pavo package^36^ in R ver. 4.3.0^37^ and the difference in chromatic (∆chrom) and achromatic (∆achrom) JNDs were calculated using the ‘coldist’ function. We used a Weber fraction of 0.1 and estimated the relative abundance of photoreceptor types as 1 UV: 1 SWS: 6 LWS for both species.

### Experiment 1 design and procedure

**Fig. 1:**
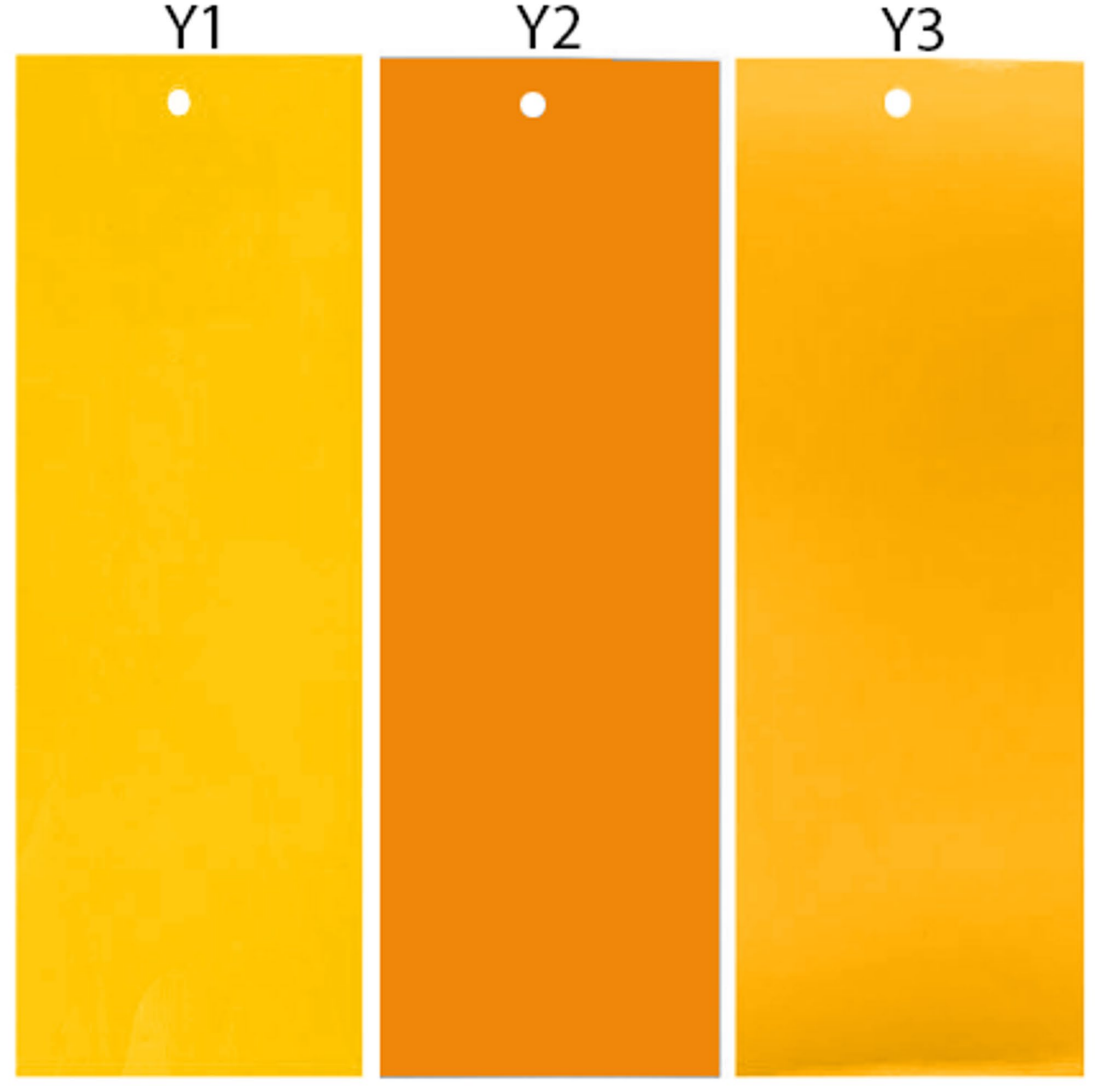
Colour treatments used for experiment 1, designed to test the effect of BG ratio and luminance on trap performance.

The goal of experiment 1 was to assess whether BG ratio, luminance, or both affect performance of yellow-orange sticky traps. We identified a bright yellow colour (Y3) that could be compared to a colour (Y1) with a higher BG ratio, but similar lightness to Y3, and to a darker colour (Y2), with a similar BG ratio for both the GWF and WFT visual system (Supplemental Table S1, Fig. 1). Therefore, if a more negative BG ratio improves sticky card performance, Y1 should increase capture relative to Y2 and Y3. If increased lightness improves sticky card performance, Y1 and Y3 should both capture significantly more individuals than Y2. If BG and lightness have an additive or interactive effect, then we predict capture to be: Y1 > Y3 > Y2. Selected colours were printed using the same paper and printer settings as for colour swatch production and cut to 100 × 245 mm. JND values for Δchrom and Δachrom for all comparisons are shown in Supplemental Table S2.

Field trials took place in three commercial polytunnels growing peppers (*Capsicum annuum)* in Antalya, Türkiye (37.041110, 30.853910) on 25 September 2023. Polytunnel 1 and 2 were approximately 40 m × 80 m and had an area of 0.30 and 0.35 hectares, respectively. Polytunnel 3 was 50 m × 80 m and had an area of 0.4 hectares. Each polytunnel had a minimum of 10 rows of crops, spaced 2.5 m apart, and was approximately 30 m long. Five rows in each polytunnel were randomly selected for trials. On each row, two traps per treatment were hung approximately 10 cm above the crop canopy in a random order with 5 m between traps for a total of N = 30/treatment across all three polytunnels. Traps were taken down after a 24-h period, and the number of GWF and WFT on each trap was counted.

After traps were hung, we photographed n = 3 cards per polytunnel for each treatment to assess Δchrom and Δachrom between colour swatch photography and measurements taken in the field. Photographs of one Y2 card was removed from analysis due to problems with image capture, for a total N = 9 for Y1 and Y3 and N = 8 for Y2. Photographs of traps included a 2% and 75% grey standard and were taken in the visible and UV spectra, and then aligned to create a multispectral image using the same methods described above. For each photo, we calculated the quantal catch for GWF and WFT, allowing us to calculate JNDs between colour swatch photography conducted in direct sunlight in a different location and measurements taken from photographs taken in the testing environment.

### Experiment 2 design and procedure

**Fig. 2:**
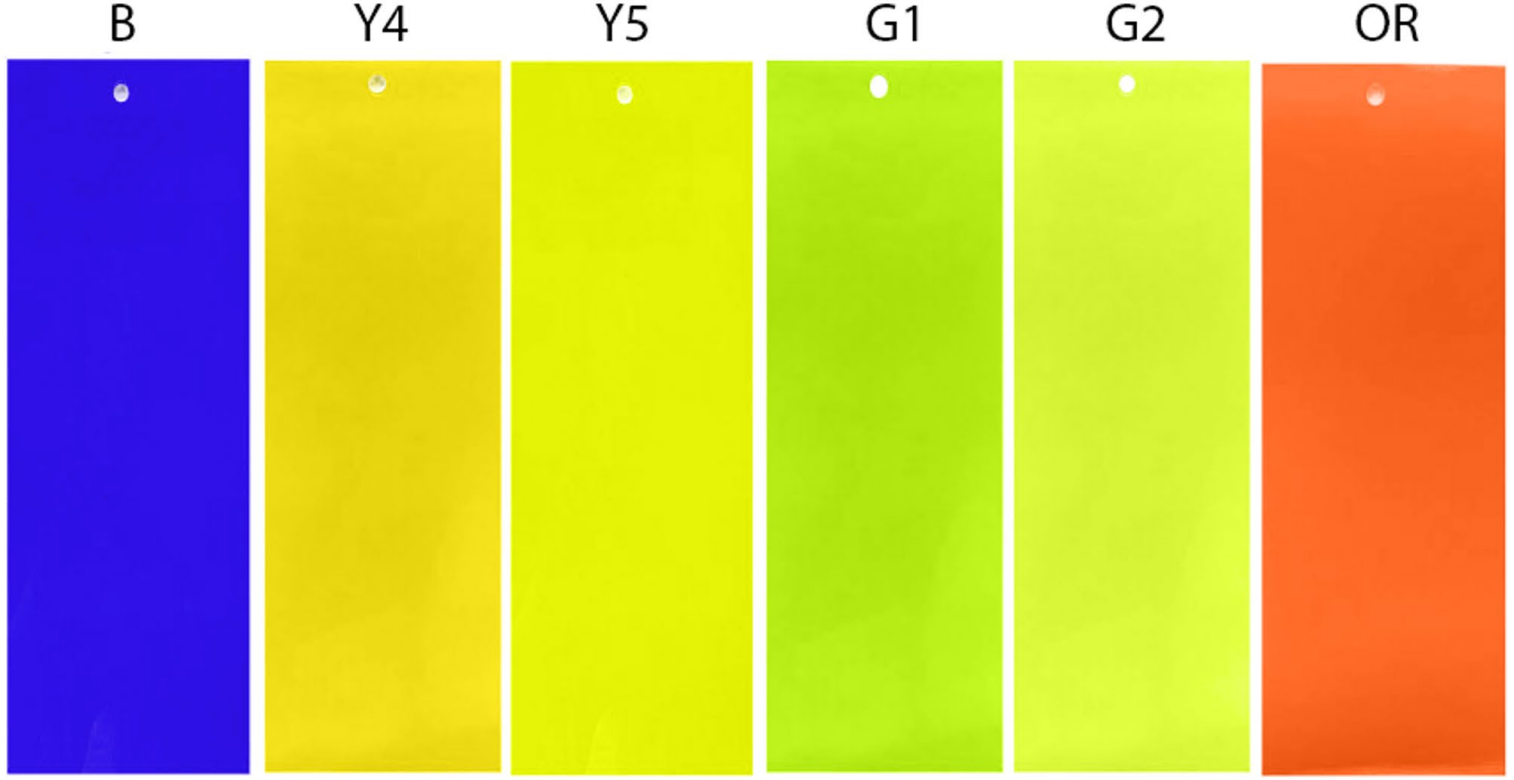
Colour treatments used for multi-pest capture experiments for experiment 2.

The goal of experiment 2 was to determine whether visual modelling approaches could assist design of sticky trap colour for targeting multiple pest species. For this experiment, we selected five colours ranging from yellow-green to orange-red that differed in BG ratio and luminance. These included two yellow traps (Y4, Y5), two yellow-green traps (G1, G2), and one orange-red trap (OR). We also included one blue (B) sticky trap since blue colours are known to be attractive to WFT (Supplemental Table S3; Fig. 2). All colours were different from those tested in experiment 1. Colours selected are predicted to be attractive to GWF and WFT based on their BG ratio, luminance, or a combination of both. If optimising BG ratio is most effective for multi-pest capture, then we predicted that Y4 and Y5 sicky traps would catch more insects than G1, G2, or OR traps. If optimising trap luminance if most effective, then G2 should capture more insects, followed by Y4, Y5, and G1, with OR capturing the least relative to the other colours. Lastly, if there is an effect of both BG ratio and luminance, then the green colours, particularly G2 should perform better than expected based on BG ratio alone. See Supplemental Table S4 for JND values between all colour comparisons for experiment 2.

Field trials took place between 28 and 30 September 2023 in the same commercial pepper polytunnels as in experiment 1. Selected colours were printed using the same paper and printer setting as used in colour swatch production and cut to 100 × 245 mm. One trap per treatment was hung within the same row of crops, approximately 10 cm above the crop canopy and with 2.5 m between each trap. Colours were randomized within rows, and this was repeated in five rows of crops across three polytunnels, for a total of N = 30/colour tested. One Y2 and two G2 traps were unable to be recovered from the field, giving N = 29 and N = 28 for these colours, respectively. To increase insect capture after an initial 24-h period, traps were left for a total of 48 h, after which the number of GWF and WFT on each trap was counted.

### Statistical analysis

All data analysis was done using R ver. 4.3.0^37^. We used a modelling approach to test the effect of different components of sticky trap colour on pest capture using the package glmmTMB^38^. We compared model AICs to assess whether the random effects of polytunnel and row number improved model performance and used the DHARMa package^39^ to select which distribution best modelled pest capture without violating model assumptions. For models that included more than one fixed effect, we assessed whether the inclusion of each term or interaction between terms significantly improved model fit using a backward model selection procedure. After selection of the final model structure, the model was run using restricted maximum likelihood (REML) estimation and post-hoc analyses were conducted using the emmeans package^40^. We used the MuMIN package to estimate R^2^ values of final models using a trigamma function^41^.We assessed normality and uniformity of residuals, dispersion, and heteroscedasticity to ensure final models do not violate standard assumptions using DHARMa’s built-in diagnostic plots.

#### Experiment 1 analysis

We assessed the effect of sticky trap colour (factor with 3 levels: Y1, Y2, Y3) on GWF and WFT catch. For the GWF dataset, we found that a negative binomial distribution best fit the data structure and comparison of model AICs indicated that the inclusion of polytunnel as a random effect improved fit over a model lacking random effects (Table S5). For the WFT dataset, a zero-inflated negative binomial distribution was used to model the effect of trap colour on WFT catch. Comparisons of model AICs indicated that the inclusion of random effects did not improve model fit so these were removed from the final model for WFT (Table S6).

While analysis of JNDs indicated that predicted BG ratios and luminance are comparable between colour swatches and printed traps in testing conditions (see Results), variation in trap colour due to differences in printing or due to interactions with light environment may alter the categorical predictions of trap colour. Therefore, in addition to assessing trap colour as a categorical factor for pest catch, we also analysed trap performance based on BG ratios and luminance values measured in the field. To achieve this, we first calculated the BG ratio and luminance for photographs of sticky traps taken in the field using the micaToolbox. We then averaged BG ratio and luminance values measured from photographs for sticky cards of the same colour category (Y1, Y2, Y3) within each polytunnel and applied this average to all cards of that colour within the same polytunnel. To determine the relationship between BG ratio and luminance on trap catch, we began with a full model that considered measured BG ratio, measured luminance, and their interaction on sticky trap catch. Data exploration for GWF suggested a non-linear effect of model terms on trap catch; we therefore included squared components of BG ratio and luminance in the initial model structure. Comparison of model AICs indicated that the inclusion of random effects did not improve model fit for either GWF or WFT so random terms were removed from further model selection steps.

For GWF, we found that a negative binomial error distribution best fit the data, and backward model selection resulted in a final model that included measured BG ratio, its quadratic component, and measured luminance on sticky trap catch (Table S7). For WFT, data fit a negative binomial model with a zero-inflation component for categorical trap colour. Backwards model selection indicated no significant effect of measured luminance in sticky trap performance, resulting in a final model that included only the effect of BG ratio on WFT catch (Table S8).

#### Experiment 2 analysis

To determine whether visual modelling can optimise colour for simultaneous monitoring of GWF and WFT, we assessed the effect of sticky trap colour (factor with 7 levels: B, Y4, Y5, G1, G2, OR), species (factor with 2 levels: GWF, WFT) and their interaction on sticky trap catch. Comparison of model AICs indicated the inclusion of crop row as a random effect improved model fit, and the data best fit a zero-inflated negative binomial distribution, with sticky trap colour in the zero-inflation component. Backwards model selection suggested a significant interaction between sticky trap colour and species on trap catch, so the final model included trap colour, species, and their interaction as fixed effects (Table S9).

We next ran separate models for GWF and WFT to determine which visual elements impact trap attractiveness within species, with BG ratio, luminance, and their interaction as possible fixed effects. Blue (B) sticky traps were excluded from this modelling given the large difference in BG ratio between this and the other colours tested. For GWF, we fitted a negative binomial model with crop row as the random effect component. Analysis of the fixed effect structure indicated an additive effect of BG ratio and luminance on GWF catch (Table S10). For WFT, comparisons of model AICs suggested the inclusion of random effects did not improve model fit, so random terms were therefore excluded from the final model. Backwards model selection for WFT resulted in a final model consisting only of BG ratio on sticky trap performance with a zero-inflation component for trap colour (S11).

## Results

### Experiment 1

Comparisons of predicted quantal catch for GWF and WFT from colour swatch photography and from photographs of sticky traps in field conditions showed that chromatic and achromatic properties of sticky traps were comparable across environments (i.e. JND < 3; Table 1).

**Table 1:** Mean ± standard deviation of the difference in chromatic (Δchrom) and achromatic (Δachrom) just noticeable differences (JNDs) for colour traps photographed in Swansea, UK and Antalya, Türkiye for the greenhouse whitefly (GWF) and Western flower thrips (WFT) visual systems. JNDs < 3 are generally considered to be poorly discriminable.

Colour	GWF		WFT	
	Δchrom	Δachrom	Δchrom	Δachrom
Y1	2.327 ± 1.313	1.251 ± 1.227	2.513 ± 1.401	1.087 ± 1.035
Y2	1.735 ± 0.906	2.027 ± 1.522	2.011 ± 0.998	1.634 ± 1.179
Y3	0.718 ± 0.307	2.180 ± 1.297	0.878 ± 0.356	1.946 ± 1.225

**Fig. 3:**
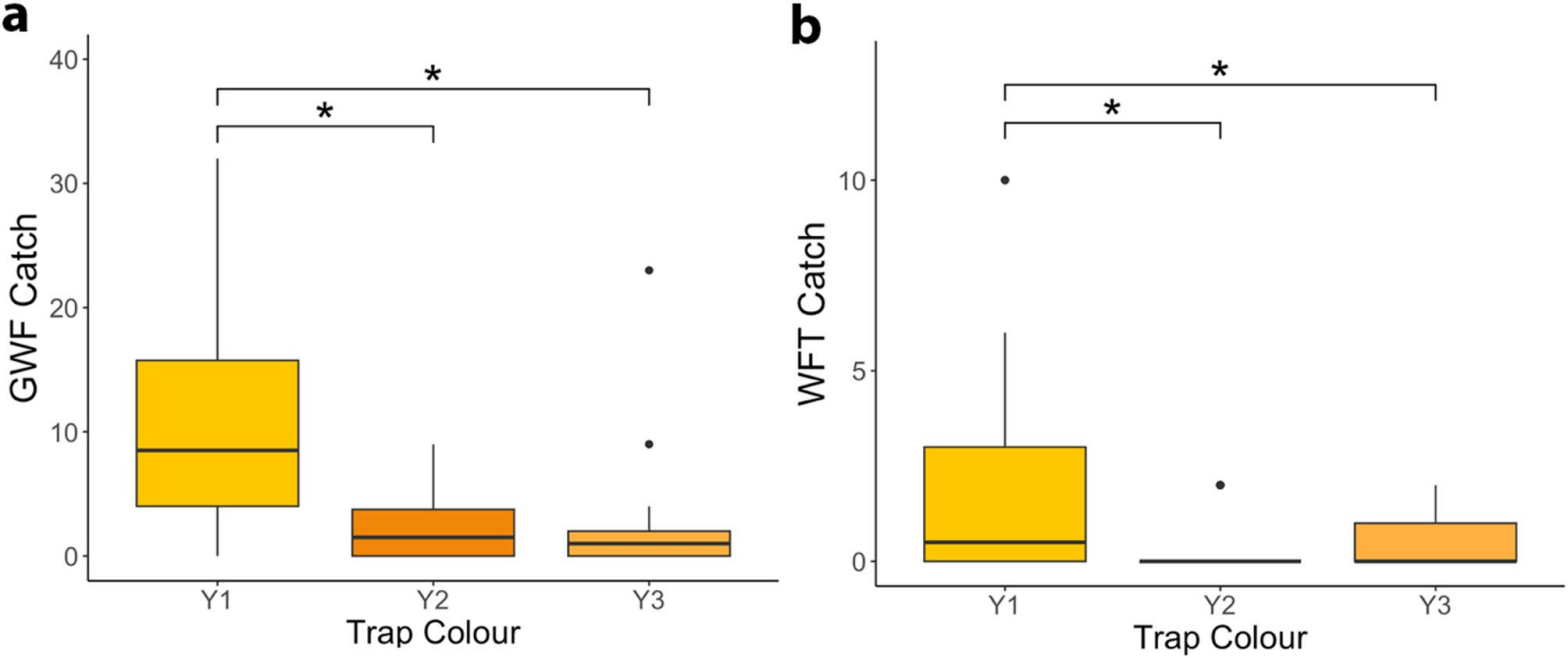
Trap catch for (**a**) greenhouse whitefly (GWF) and (**b**) Western flower thrips (WFT) on yellow sticky traps differing in predicted blue-green (BG) ratio and luminance. BG ratio represents the intensity of the opponent response between the blue (SWS) and green (LWS) photoreceptor cells and is greatest for Y1 and approximately equal for Y2 and Y3 for both species. Predicted luminance is greatest and approximately equal for Y1 and Y3 relative to Y2 for both species (see Supplemental Table S1). An asterisk (*) indicates a significant difference between colours following Tukey correction for multiple comparisons.

Results showed a significant difference between yellow colours tested for GWF catch (χ^2^ = 67.340, df = 2, *p* < 0.001; Fig. 3a). Post-hoc tests with a Tukey correction show that the yellow colour with both high BG ratio and luminance (Y1) caught significantly more GWF (mean ± se = 10.267 ± 1.425) compared to Y2 (2.000 ± 0.407), which has a lower BG ratio and luminance than Y1 (Y1—Y2: Z = 6.210,* p* < 0.001). Y1 also caught significantly more GWF than Y3 (2.067 ± 0.799) (Y1 – Y3: Z = 6.672, *p* < 0.001), which has similar luminance but a lower BG ratio than Y1. There was no significant difference in GWF caught between Y2 and Y3 (Y2 – Y3: Z = -0.858, *p* = 0.666), despite large differences in predicted luminance between these two colours (Supplemental Tables S1, S2).

There was also a significant difference in WFT capture between yellow sticky traps (χ^2^ = 34.081, df = 2, *p* < 0.001; Fig. 3b). Results of post-hoc tests with a Tukey correction showed Y1 (mean ± se = 2.066 ± 0.529) caught significantly more WFT compared to Y2 (0.33 ± 0.138) (Y1 – Y2: Z = 3.267, *p* = 0.003), which had both a lower BG ratio and luminance relative to Y1. Y1 also caught significantly more WFT than Y3 (0.633 ± 0.140), which is similar in luminance to Y1 but has a lower BG ratio (Y1–Y3: Z = 2.719, *p* = 0.018). There was no significant difference between Y2 and Y3 (Z = -1.320, *p* = 0.384), which had similar BG ratio, but differed in luminance (Supplemental Tables S1, S2).

**Fig. 4:**
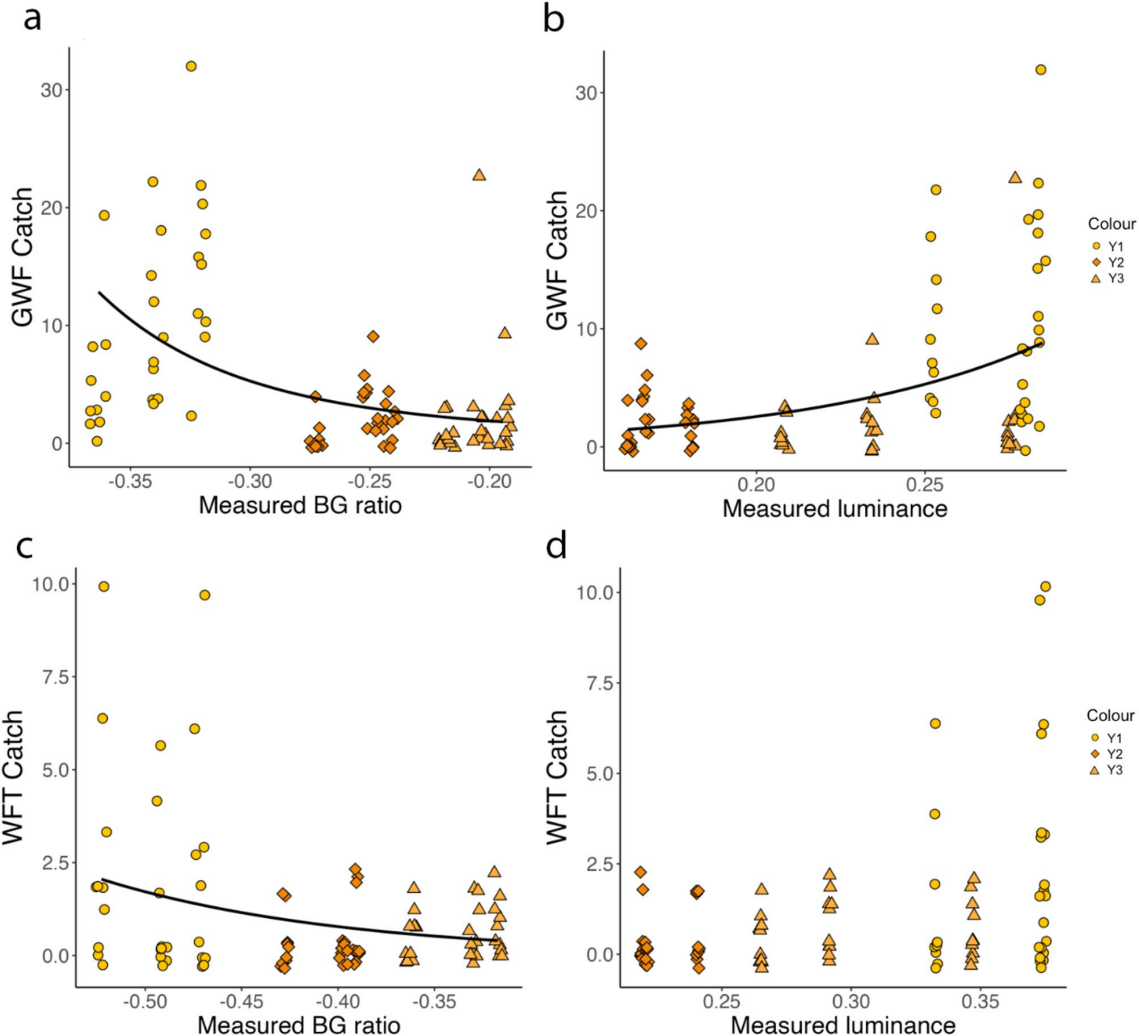
Blue-green (BG) ratio and luminance of yellow-orange sticky traps measured in the field and insect capture. (**a**) BG ratio and (**b**) luminance measured from sticky traps in the field showed a significant relationship to the number of greenhouse whitefly (GWF) captured on yellow sticky traps. Conditional R^2^ for the final model for GWF, including BG ratio, its squared component, and luminance was 0.26. (**c**) There is a significant relationship between measured BG ratio and trap catch for Western flower thrips (WFT), (**d**) but no significant relationship was observed between trap luminance and WFT catch. R^2^ for the final model for WFT was 0.19. Trap colour is depicted in plots via colour and shape, with Y1 shown as circles, Y2 as diamonds, and Y3 as triangles. (**a**–**c**) Plotted trendlines represent a negative binomial regression fit.

Analysing insect catch using measured BG ratio and luminance resulted in similar outcomes to categorical comparison of trap colours. For GWF, results showed a significant effect of measured BG ratio (χ^2^ = 18.194, df = 1, *p* < 0.001) and its quadratic component (χ^2^ = 16.056, df = 1, *p* < 0.001) on insect catch, with more extreme BG ratios catching more GWF relative to values closer to zero (Fig. 4a). There is also a significant effect of sticky trap luminance measured in the field on predicted GWF catch, with a positive correlation between trap luminance and GWF catch (χ^2^ = 26.239, df = 1, *p* < 0.001; Fig. 4b). Conditional R^2^ = 0.26 for the final model, including BG ratio, its quadratic component, and luminance. For WFT, the only term remaining after model reduction was BG ratio, with a significant decrease in WFT caught as the BG ratio became closer to zero (χ^2^ = 11.233, df = 1, *p* < 0.001; Fig. 4c,d). Conditional R^2^ of the final model for WFT capture was 0.19.

### Experiment 2

**Fig. 5:**
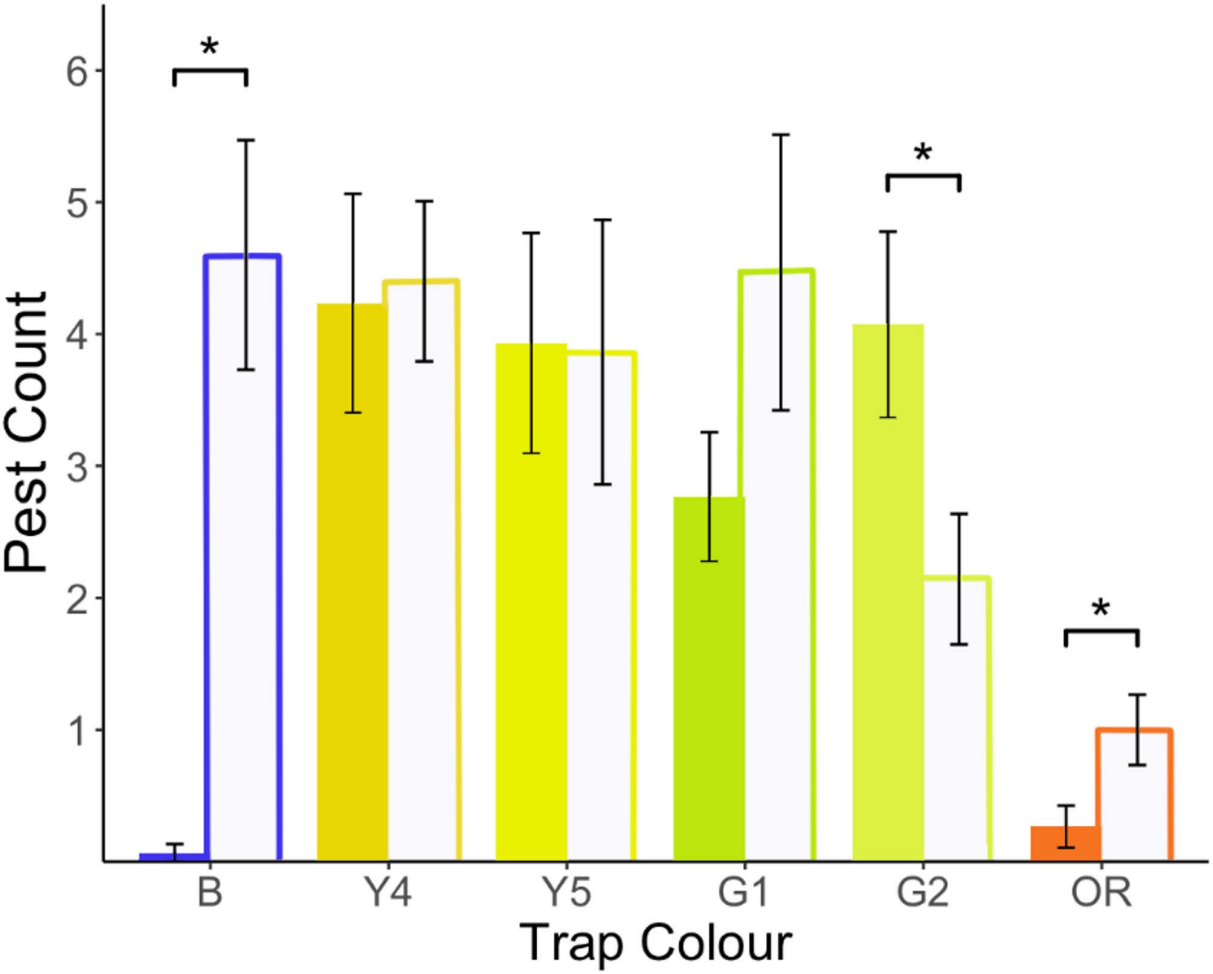
Pest catch on different coloured sticky traps for greenhouse whitefly (GWF) (filled bars) and Western flower thrips (WFT) (unfilled bars). Trap colours tested are blue (B), two yellow (Y4, Y5), two yellow-green (G1, G2), and an orange-red (OR). Bar/outline colour represents sticky trap colour, with data for GWF shown in filled and WFT shown in outlined bars. An asterisk (*) indicates a significant difference between species.

There was a significant interaction between sticky trap colour and insect species on overall trap catch (χ^2^ = 12.838, df = 5, *p* = 0.025). Post-hoc comparisons showed that significantly more WFT were caught on B (Z = −4.977, *p* < 0.001) and OR (Z = −2.596,* p* = 0.009), and significantly fewer on G2 (Z = 2.202,* p* = 0.028), relative to GWF. The G1 sticky trap tended to catch more WFT than GWF, but the difference between species was not significant (Z = −1.890, *p* = 0.059) (Fig. 5). Within species, there were significant differences in trap performance based on colour, with B and OR performing significantly worse compared to yellow (Y4, Y5) and yellow-green (G1, G2) colours for GWF (Table 2, Supplemental Table S12). For WFT, B cards caught similar number of individuals as both yellows (Y4, Y5) and one yellow-green tested (G1), with low WFT catch for OR and intermediate catch for G2 (Table 2, Supplemental Table S12).

**Table 2:** Mean ± standard error of greenhouse whitefly (GWF) and Western flower thrips (WFT) caught on different coloured sticky traps after 48 h. Traps include a blue (B), two yellows (Y4, Y5), and yellow-greens (G1, G2), and an orange-red (OR) colour. Significant differences within species in the number of individuals caught are indicated by letters. See Supplemental Table S12 for p-values for all pair-wise comparisons.

Colour	GWF		WFT	
B	0.067 ± 0.067	*(a)*	4.600 ± 0.870	*(a)*
Y4	4.233 ± 0.830	*(b)*	4.400 ± 0.607	*(a)*
Y5	3.931 ± 0.835	*(b)*	3.862 ± 1.003	*(a)*
G1	2.767 ± 0.488	*(b)*	4.467 ± 1.046	*(a)*
G2	4.071 ± 0.705	*(b)*	2.143 ± 0.495	*(ab)*
OR	0.267 ± 0.159	*(a)*	1.000 ± 0.267	*(b)*

**Fig. 6:**
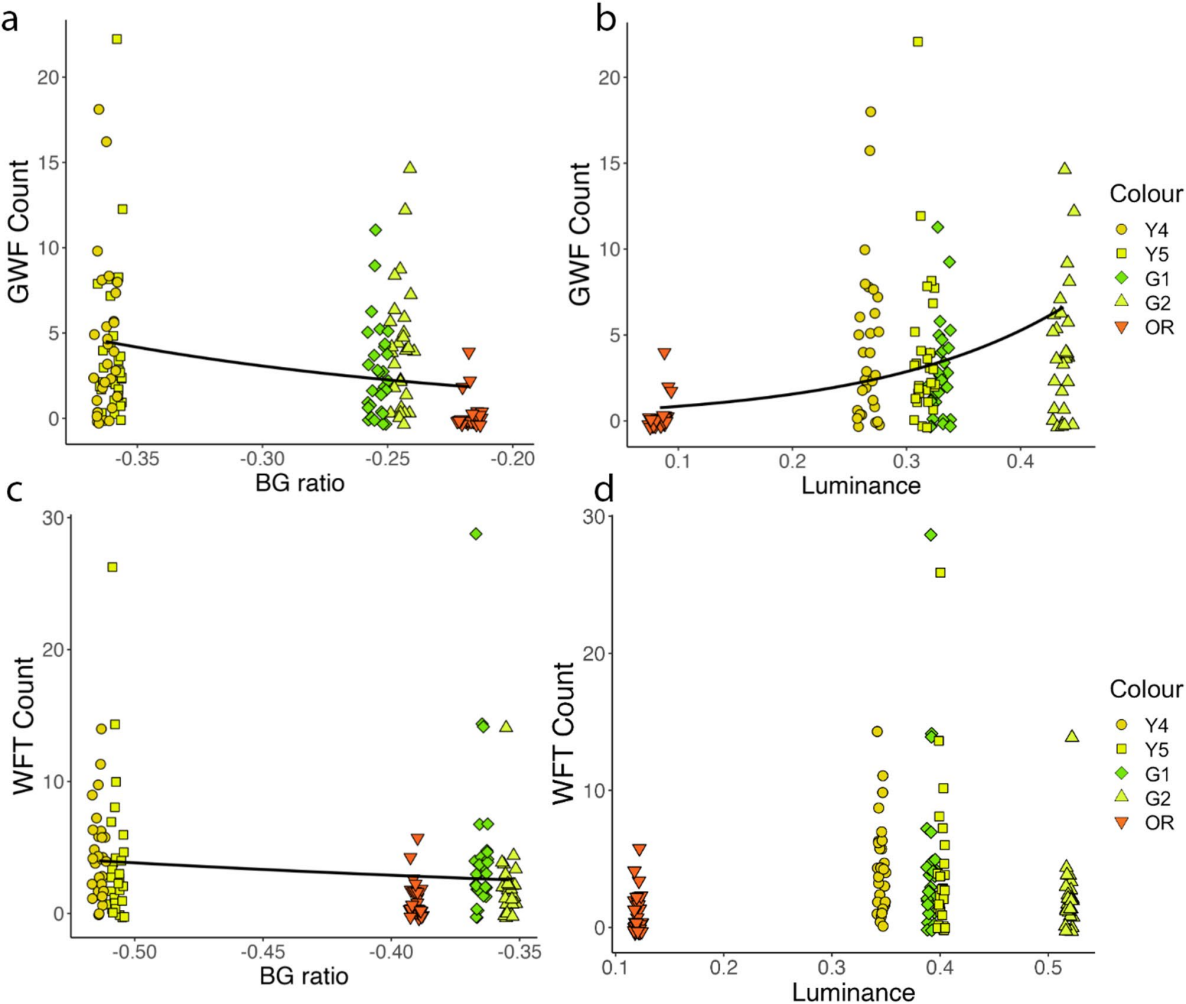
Relationship between blue-green (BG) ratio, luminance, and trap catch for greenhouse whitefly (GWF) and Western flower thrips (WFT) for yellow-green to orange-red colours. (**a**, **c**) There is a significant correlation between BG ratio and pest capture for GWF and WFT, with colours that are more highly stimulating to the green relative to blue photoreceptor (smaller values) performing better than traps with a lower opponent response (larger values). (**b**) There is a positive correlation between trap luminance and GWF catch, (**d**) but no significant relationship between luminance and WFT catch. (**a**–**c**) Plotted trendline represents a negative binomial regression fit. Conditional R^2^ for the model showing the effect of BG ratio and luminance on GWF capture was 0.33 and for the WFT model, which included only BG ratio, was 0.02.

Modelling results for GWF indicate a significant additive effect of BG ratio (χ^2^ = 20.724, df = 1, *p* < 0.001) and luminance (χ^2^ = 29.811, df = 1, *p* < 0.001) on trap capture (Fig. 6a,b). Conditional R^2^ for this model was 0.33. For WFT, the final model included a significant effect of BG ratio (χ^2^ = 3.947, df = 1, *p* = 0.047) on trap catch, with a conditional R^2^ of 0.02 (Fig. 6c). Model selection suggested no significant effect of luminance on WFT capture (Fig. 6d).

## Discussion

Previous work has shown that using visual modelling to predict the BG opponent response can optimise WFT catch to blue coloured sticky traps^19^. Here, we show that modelling BG ratio can also predict performance of yellow sticky traps for both WFT and GWF. We also find that achromatic luminance cues affect trap catch for GWF but not WFT. Importantly, modelled chromatic and achromatic trap measures in one environment (Swansea, UK) translated to modelled chromatic and achromatic measures in the testing environment (Antalya, Türkiye), suggesting that visual modelling approaches are robust and have utility across at least some degree of variation in testing environment. Overall, our results provide support for the use of visual modelling in the efficient design of pest traps^16^ and show that BG opponent responses produced via visual modelling offer a principled approach for targeting monitoring towards multiple pest species.

While previous studies typically consider preference for blue or yellow colours in GWF and WFT, often with conflicting results, in the current study we focus on the specific properties of colour (i.e., BG ratio and luminance) that impact attractiveness to yellow colours. Previous work has found that optimising the BG ratio based to maximize stimulation of the blue relative to the green photoreceptor increased trap catch for blue sticky traps in WFT^19^. Our results show that optimising the BG ratio in the opposite direction, by maximizing stimulation of the green relative to the blue photoreceptor, also improved trap performance for WFT capture and that this principal also applies to other pest species (i.e., GWF).

While preference for blue is generally considered stronger for WFT, yellow sticky traps have been suggested previously as a potential method for monitoring both GWF and WFT simultaneously^12^. However, the specific visual properties of yellow sticky traps that allow for effective simultaneous monitoring of GWF and WFT had not been previously evaluated in detail. Our results showed differences in preference for several yellow colours tested on both WFT and GWF (Fig. 5). Trap performance was related to the BG ratio in both species, with yellow colours that maximize the opponent response increasing attractiveness compared to other colours and performing as well as the blue sticky trap in WFT capture (Table 2; Fig. 5). Achromatic luminance cues also appear to shape trap attractiveness for GWF, in line with previous studies utilising luminous LED traps^25^. However, our results suggest that achromatic component of traps does not affect WFT capture, at least over the range tested. Previous studies in WFT have found an effect of trap brightness for colours in the blue-violet range, but no effect in the green-yellow range^42^, in line with our results. Overall, this suggests that while yellow colours can be effective for monitoring both GWF and WFT, performance of yellow colours is not uniform across the spectrum and the factors that impact trap success can vary between species. Yellow colours are more generally attractive across a wide number of insect species, including some species of beneficial insects^2,15^. For example, yellow was more successful than blue or white traps at collecting several species of Hymenoptera, which are beneficial for plant pollination^43,44^. Our results indicate that visual modelling can aid navigation of the trade-off between attracting target pests while reducing unintended capture of non-target insect species, for example by determining the BG ratio for target pests and co-occurring beneficials. Future studies following the methodologies we outline in the current study will be valuable in demonstrating whether visual modelling can better tailor sticky traps for only intended pest targets.

We also showed that chromatic and achromatic properties of selected colours in experiment 1 are similar across various environments, specifically between Swansea, UK where initial visual modelling took place and Antalya, Türkiye, where behavioural testing took place. Variation in the location, timing, and weather conditions during photography are all possible factors that could impact the replicability of visual modelling. Variation between the thickness, age, or type of material used in polytunnel construction can also have an impact on light environment, and therefore visual perception^45^. Despite this, we found that JNDs between colour swatches photographed in Swansea, UK and from photographs of printed sticky traps in the field were on average < 3 (Table 1), indicating visual differences between stimuli are likely not discriminable under normal viewing conditions by the target species. While modelling results are likely not generalizable when there are large differences in transmission properties of certain materials or differences in lighting source (e.g., natural sunlight compared to artificial lighting), our results suggest that visual modelling can reliably predict performance across typical conditions used for commercial crop growers. Further, visual modelling can easily incorporate the effect of different environmental illumination on colour perception^29^, meaning that prior knowledge of the light environment can further improve predications based on modelling results.

Visual modelling can be used to further optimise trap design by aiding design of traps with internal contesting patterns. Previous studies have shown that adding a highly-contrasting yellow flower pattern to blue sticky cards significantly increases trap catch relative to solid blue cards for WFT^19^. Similarly, adding contrasting black patterns to yellow traps also increased trap catch for GWF^46^, while reducing the level of contrast between plants and the background reduced settling relative to plants with higher contrast to the background^47^. The increased attraction of GWF and WFT to stimuli with higher internal contrast, paired with our results showing that optimised yellow colours perform as well as blues for WFT (Table 2) and are simultaneously attractive to both target species (Fig. 5), suggests that development of contrasting blue-yellow traps could further increase trap effectiveness for both pest species.

Overall, our study provides support for the use of visual modelling in the monitoring of multiple pest species. Accounting for visual properties of coloured sticky cards explains differences in preferences for both GWF and WFT, with the opponent response of colours corresponding with trap catch. This may further provide insights into differences in colour preferences commonly reported between studies in WFT, with differences between shades of yellow tested varying in their relative attractiveness to one another and to a blue coloured card. We showed that predicted and measured visual properties of sticky traps are consistent across polytunnels, despite differences in environments in which colour properties were predicted and in which testing occurred. In all, these results support the reliability of visual modelling in trap design and its prospect for improving of pest monitoring and management.

## Supplementary Information

Supplementary Information.

## Data Availability

Datasets analysed for the current study are available on the Dryad repository https://doi.org/10.5061/dryad.h9w0vt4rf.
