# Supplementary material for: Visual modelling can optimise sticky trap design for simultaneous monitoring of multiple species of insect pests
Source: Sci Rep. 2025 May 19;15:17280. doi: 10.1038/s41598-025-01954-8 (PMC12089412; doi:10.1038/s41598-025-01954-8)
Supplement: Supplementary file 1 — Supplementary Information. [file 41598_2025_1954_MOESM1_ESM.docx]

**Supplemental Table S1.** Predicted blue‒green (BG) ratio and luminance of sticky straps for the visual system of greenhouse whitefly (GWF) and Western flower thrips (WFT) for experiment 1. Yellow-orange colours (Y1, Y2, Y3) differ in either BG ratio or luminance. Commercial Agrisense cards are shown for comparison.

|  | GWF | | WFT | |
| --- | --- | --- | --- | --- |
| Colour | BG ratio | Luminance | BG ratio | Luminance |
| Y1 | -0.29 | 0.30 | -0.43 | 0.38 |
| Y2 | -0.19 | 0.21 | -0.32 | 0.26 |
| Y3 | -0.19 | 0.29 | -0.31 | 0.36 |
| Agrisense Commercial Yellow | -0.27 | 0.32 | -0.39 | 0.38 |

**Supplemental Table S2.** Chromatic (Δchrom) and achromatic (Δachrom) just noticeable differences (JNDs) between coloured sticky traps used in experiment 1 given the visual system of greenhouse whitefly (GWF) and Western flower thrips (WFT). JNDs < 1 are considered below threshold for discrimination.

| Colour | GWF | | WFT | |
| --- | --- | --- | --- | --- |
|  | Δchrom | Δachrom | Δchrom | Δachrom |
| Y1 - Y2 | 2.25 | 3.64 | 2.31 | 3.73 |
| Y1 - Y3 | 1.82 | 0.14 | 2.03 | 0.54 |
| Y2 - Y3 | 0.47 | 3.50 | 0.40 | 3.19 |

**Supplemental Table S3.** Predicted blue‒green (BG) ratio and luminance of sticky straps for the visual system of greenhouse whitefly (GWF) and Western flower thrips (WFT) for experiment 2. Colours included a blue (B), two yellows (Y4, Y5), two yellow-green (G1, G2), and an orange-red (OR).

|  | GWF | | WFT | |
| --- | --- | --- | --- | --- |
| Colour | BG ratio | Luminance | BG ratio | Luminance |
| B | 0.17 | 0.23 | 0.31 | 0.17 |
| Y4 | -0.36 | 0.27 | -0.51 | 0.35 |
| Y5 | -0.36 | 0.32 | -0.51 | 0.40 |
| G1 | -0.25 | 0.33 | -0.36 | 0.39 |
| G2 | -0.24 | 0.44 | -0.35 | 0.52 |
| OR | -0.22 | 0.08 | -0.39 | 0.12 |

**Supplemental Table S4.** Chromatic (Δchrom) and achromatic (Δachrom) just noticeable differences (JNDs) between coloured sticky traps used in experiment 2 given the visual system of greenhouse whitefly (GWF) and Western flower thrips (WFT). JNDs < 1 are considered below threshold for discrimination.

| Colour | GWF | | WFT | |
| --- | --- | --- | --- | --- |
|  | Δchrom | Δachrom | Δchrom | Δachrom |
| B - Y1 | 7.94 | 6.83 | 4.68 | 1.38 |
| B - Y2 | 8.35 | 8.33 | 5.14 | 3.08 |
| B - G1 | 5.76 | 8.08 | 3.28 | 3.51 |
| B - G2 | 5.51 | 10.89 | 3.19 | 6.35 |
| B - OR | 5.83 | 3.71 | 5.05 | 10.17 |
| Y1 - Y2 | 0.82 | 1.50 | 0.91 | 1.70 |
| Y1 - G1 | 2.47 | 1.25 | 1.96 | 2.13 |
| Y1 - G2 | 2.95 | 4.07 | 2.50 | 4.96 |
| Y1 - OR | 5.99 | 10.54 | 6.33 | 11.56 |
| Y2 - G1 | 3.15 | 0.25 | 2.79 | 0.43 |
| Y2 - G2 | 3.65 | 2.56 | 3.35 | 3.26 |
| Y2 - OR | 6.79 | 12.04 | 7.22 | 13.26 |
| G1 - G2 | 0.51 | 2.82 | 0.56 | 2.83 |
| G1 - OR | 3.85 | 11.78 | 4.48 | 13.69 |
| G2 - OR | 3.35 | 14.60 | 3.92 | 16.52 |

**Supplemental Table S5.** Final model, model fit metrics, and model output for analysis of greenhouse whitefly (GWF) caught on three different colour cards.

| **GWF Count ~ Colour + (1\|Tunnel), family = "nbinom1"** | | | | |
| --- | --- | --- | --- | --- |
| Model Fit Metrics: | | | | |
| AIC | BIC | Log Likelihood | Deviance | DF. Resid. |
| 424.7 | 437.2 | -207.4 | 414.7 | 85 |
| Fixed Component: | | | | |
| Term | Estimate | Std. Error | Z-value | p-value |
| (Intercept) | 2.23 | 0.36 | 6.28 | <0.001 |
| ColourY2 | -1.45 | 0.23 | -6.21 | <0.001 |
| ColourY1 | -1.71 | 0.26 | -6.67 | <0.001 |
| Random Effects: | | | | |
| Group | Variance | Std. Dev. |  |  |
| Tunnel (Intercept) | 0.34 | 0.58 |  |  |

**Supplemental Table S6.** Final model, model fit metrics, and model output for analysis of western flower thrips (WFT) caught on three different colour cards.

| **WFT Count ~ Colour, ziformula = ~Colour, family = "nbinom2"** | | | | |
| --- | --- | --- | --- | --- |
| Model Fit Metrics: | | | | |
| AIC | BIC | Log Likelihood | Deviance | DF. Resid. |
| 227.3 | 244.8 | -106.6 | 213.3 | 83 |
| Fixed Component (Conditional Model): | | | | |
| Term | Estimate | Std. Error | Z-value | p-value |
| (Intercept) | 1.35 | 0.19 | 6.98 | <0.001 |
| ColourY2 | -1.02 | 0.53 | -1.94 | 0.05 |
| ColourY1 | -1.81 | 0.31 | -5.79 | <0.001 |
| Fixed Component (Zero-inflation Model): | | | | |
| (Intercept) | -0.14 | 0.41 | -0.35 | 0.73 |
| ColourY2 | 1.29 | 0.71 | 1.81 | 0.07 |
| ColourY1 | -17.61 | 8112.48 | 0.00 | 1.00 |

**Supplemental Table S7.** Final model, model fit metrics, and model output showing the effect of measured blue-green (BG) ratio and luminance (lum) on greenhouse whitefly (GWF) capture. Measured BG and lum are taken from photographs of sticky traps hanging in commercial polytunnels.

| **GWF Count ~ measured BG+measured Lum +I(measured BG^2), family = "nbinom1"** | | | | |
| --- | --- | --- | --- | --- |
| Model Fit Metrics: | | | | |
| AIC | BIC | Log Likelihood | Deviance | DF. Resid. |
| 420.9 | 433.4 | -205.5 | 410.9 | 85 |
| Fixed Component: | | | | |
| Term | Estimate | Std. Error | Z-value | p-value |
| (Intercept) | -17.16 | 3.72 | -4.61 | <0.001 |
| mBG | -107.89 | 25.30 | -4.27 | <0.001 |
| mLum | 14.23 | 2.78 | 5.12 | <0.001 |
| I(mBG^2) | -182.59 | 45.57 | -4.01 | <0.001 |

**Supplemental Table S8.** Final model, model fit metrics, and model output showing the effect of measured blue-green (BG) ratio and luminance (lum) on western flower thrips (WFT) capture. Measured BG and lum are taken from photographs of sticky traps hanging in commercial polytunnels.

| **WFT Count ~ measured BG, ziformula = ~Colour , family = "nbinom1"** | | | | |
| --- | --- | --- | --- | --- |
| Model Fit Metrics: | | | | |
| AIC | BIC | Log Likelihood | Deviance | DF. Resid. |
| 229.2 | 244.2 | -108.6 | 217.2 | 84 |
| Fixed Component (Conditional Model): | | | | |
| Term | Estimate | Std. Error | Z-value | p-value |
| (Intercept) | -3.27 | 1.31 | -2.49 | 0.01 |
| mBG | -9.02 | 2.69 | -3.35 | <0.001 |
| Fixed Component (Zero-inflation Model): | | | | |
| (Intercept) | -0.21 | 0.44 | -0.49 | 0.63 |
| ColourY2 | 1.32 | 0.70 | 1.89 | 0.06 |
| ColourY1 | -4.83 | 63.30 | -0.08 | 0.94 |

**Supplemental Table S9.** Final model, model fit metrics, and model output showing the effect of species (GWF and WFT) and colour on overall trap catch.

| **Count ~ Colour*Species + (1\|Row), ziformula = ~Colour + Species , family = "nbinom2"** | | | | |
| --- | --- | --- | --- | --- |
| Model Fit Metrics: | | | | |
| AIC | BIC | Log Likelihood | Deviance | DF. Resid. |
| 1450.3 | 1531.5 | -704.1 | 1408.3 | 333 |
| Fixed Component (Conditional Model): | | | | |
| Term | Estimate | Std. Error | Z-value | p-value |
| (Intercept) | -0.13 | 1.46 | -0.09 | 0.93 |
| ColourY4 | 1.71 | 1.47 | 1.16 | 0.25 |
| ColourY5 | 1.43 | 1.47 | 0.97 | 0.33 |
| ColourG1 | 1.17 | 1.48 | 0.80 | 0.43 |
| ColourG2 | 1.66 | 1.48 | 1.12 | 0.26 |
| ColourOR | 0.78 | 1.57 | 0.50 | 0.62 |
| SpeciesWFT | 1.81 | 1.47 | 1.23 | 0.22 |
| ColourY4:SpeciesWFT | -1.94 | 1.49 | -1.30 | 0.19 |
| ColourY5:SpeciesWFT | -1.81 | 1.49 | -1.21 | 0.23 |
| ColourG1:SpeciesWFT | -1.38 | 1.49 | -0.92 | 0.36 |
| ColourG2:SpeciesWFT | -2.62 | 1.50 | -1.75 | 0.08 |
| ColourOR:SpeciesWFT | -2.20 | 1.49 | -1.48 | 0.14 |
| Fixed Component (Zero-inflation Model): | | | | |
| (Intercept) | 2.17 | 1.00 | 2.16 | 0.03 |
| ColourY4 | -3.95 | 1.22 | -3.23 | <0.01 |
| ColourY5 | -20.49 | 3229.74 | -0.01 | 0.99 |
| ColourG1 | -4.85 | 2.05 | -2.36 | 0.02 |
| ColourG2 | -4.26 | 1.37 | -3.11 | <0.01 |
| ColourOR | 0.02 | 1.19 | 0.02 | 0.99 |
| SpeciesWFT | -3.57 | 1.10 | -3.26 | <0.01 |
| Random Effects: | | | | |
| Group | Variance | Std. Dev. |  |  |
| Row (Intercept) | 0.07 | 0.26 |  |  |

**Supplemental Table S10.** Final model, model fit metrics, and model output showing the effect of blue-green (BG) ratio and luminance (Lum) on greenhouse whitefly (GWF) capture.

| **GWF Count ~ BG+Lum+ (1\|Row), family = "nbinom1"** | | | | |
| --- | --- | --- | --- | --- |
| Model Fit Metrics: | | | | |
| AIC | BIC | Log Likelihood | Deviance | DF. Resid. |
| 606.7 | 621.6 | -298.3 | 596.7 | 142 |
| Fixed Component: | | | | |
| Term | Estimate | Std. Error | Z-value | p-value |
| (Intercept) | -3.02 | 0.75 | -4.01 | <0.001 |
| BG | -7.11 | 1.56 | -4.55 | <0.001 |
| Lum | 6.29 | 1.15 | 5.46 | <0.001 |
| Random Effects: | | | | |
| Group | Variance | Std. Dev. |  |  |
| Row (Intercept) | 0.13 | 0.36 |  |  |

**Supplemental Table S11.** Final model, model fit metrics, and model output showing the effect of blue-green (BG) on western flower thrips (WFT) capture.

| **WFT Count ~ BG, ziformula = ~Colour, family = "nbinom2"** | | | | |
| --- | --- | --- | --- | --- |
| Model Fit Metrics: | | | | |
| AIC | BIC | Log Likelihood | Deviance | DF. Resid. |
| 670.9 | 694.8 | -327.5 | 654.9 | 139 |
| Fixed Component (Conditional Model): | | | | |
| Term | Estimate | Std. Error | Z-value | p-value |
| (Intercept) | 0.23 | 0.53 | 0.43 | 0.67 |
| BG | -2.44 | 1.23 | -1.99 | 0.05 |
| Fixed Component (Zero-inflation Model): | | | | |
| (Intercept) | -21.31 | 11015.52 | 0.00 | 1.00 |
| ColourY5 | 19.34 | 11015.52 | 0.00 | 1.00 |
| ColourG1 | -0.95 | 24985.47 | 0.00 | 1.00 |
| ColourG2 | 2.63 | 13011.71 | 0.00 | 1.00 |
| ColourOR | 21.19 | 11015.52 | 0.00 | 1.00 |

**Supplemental Table S12.** Post-hoc comparisons following Tukey adjustment between coloured sticky cards for greenhouse whitefly (GWF) and Western flower thrips (WFT).

| Species = GWF | | | | | |
| --- | --- | --- | --- | --- | --- |
| contrast | estimate | SE | df | z.ratio | p.value |
| B - Y1 | -4.17432 | 0.813 | Inf | -5.132 | <.0001 |
| B - Y2 | -3.85452 | 0.673 | Inf | -5.727 | <.0001 |
| B - G1 | -2.69113 | 0.525 | Inf | -5.131 | <.0001 |
| B - G2 | -4.01743 | 0.788 | Inf | -5.1 | <.0001 |
| B - OR | -0.13935 | 0.182 | Inf | -0.766 | 0.9732 |
| Y1 - Y2 | 0.3198 | 1.05 | Inf | 0.305 | 0.9997 |
| Y1 - G1 | 1.48318 | 0.962 | Inf | 1.543 | 0.6366 |
| Y1 - G2 | 0.15689 | 1.127 | Inf | 0.139 | 1 |
| Y1 - OR | 4.03497 | 0.826 | Inf | 4.884 | <.0001 |
| Y2 - G1 | 1.16339 | 0.846 | Inf | 1.375 | 0.7422 |
| Y2 - G2 | -0.16291 | 1.03 | Inf | -0.158 | 1 |
| Y2 - OR | 3.71517 | 0.688 | Inf | 5.396 | <.0001 |
| G1 - G2 | -1.32629 | 0.94 | Inf | -1.411 | 0.7204 |
| G1 - OR | 2.55178 | 0.544 | Inf | 4.69 | <.0001 |
| G2 - OR | 3.87807 | 0.801 | Inf | 4.841 | <.0001 |

| Species = WFT | | | | | |
| --- | --- | --- | --- | --- | --- |
| contrast | estimate | SE | df | z.ratio | p.value |
| B - Y1 | 0.07993 | 1.138 | Inf | 0.07 | 1 |
| B - Y2 | 0.6089 | 1.099 | Inf | 0.554 | 0.9938 |
| B - G1 | 0.00271 | 1.146 | Inf | 0.002 | 1 |
| B - G2 | 2.33025 | 0.972 | Inf | 2.396 | 0.1574 |
| B - OR | 3.44015 | 0.919 | Inf | 3.742 | 0.0025 |
| Y1 - Y2 | 0.52897 | 0.979 | Inf | 0.54 | 0.9945 |
| Y1 - G1 | -0.07722 | 1.032 | Inf | -0.075 | 1 |
| Y1 - G2 | 2.25032 | 0.835 | Inf | 2.695 | 0.0761 |
| Y1 - OR | 3.36022 | 0.772 | Inf | 4.353 | 0.0002 |
| Y2 - G1 | -0.60618 | 0.987 | Inf | -0.614 | 0.99 |
| Y2 - G2 | 1.72135 | 0.779 | Inf | 2.211 | 0.2327 |
| Y2 - OR | 2.83125 | 0.711 | Inf | 3.984 | 0.001 |
| G1 - G2 | 2.32754 | 0.844 | Inf | 2.757 | 0.0646 |
| G1 - OR | 3.43744 | 0.782 | Inf | 4.396 | 0.0002 |
| G2 - OR | 1.1099 | 0.494 | Inf | 2.247 | 0.2163 |
